# Predictive Modeling to Study the Treatment-Shortening Potential of Novel Tuberculosis Drug Regimens, Toward Bundling of Preclinical Data

**DOI:** 10.1093/infdis/jiab101

**Published:** 2021-02-19

**Authors:** Saskia E Mudde, Rami Ayoun Alsoud, Aart van der Meijden, Anna M Upton, Manisha U Lotlikar, Ulrika S H Simonsson, Hannelore I Bax, Jurriaan E M de Steenwinkel

**Affiliations:** Department of Medical Microbiology and Infectious Diseases, Erasmus University Medical Center, Rotterdam, the Netherlands; Department of Pharmaceutical Biosciences, Uppsala University, Uppsala,Sweden; Department of Medical Microbiology and Infectious Diseases, Erasmus University Medical Center, Rotterdam, the Netherlands; Global Alliance for Tuberculosis Drug Development, New York, New York, USA; Global Alliance for Tuberculosis Drug Development, New York, New York, USA; Department of Pharmaceutical Biosciences, Uppsala University, Uppsala,Sweden; Department of Medical Microbiology and Infectious Diseases, Erasmus University Medical Center, Rotterdam, the Netherlands; Department of Internal Medicine, Section of Infectious Diseases, Erasmus University Medical Center, Rotterdam, the Netherlands; Department of Medical Microbiology and Infectious Diseases, Erasmus University Medical Center, Rotterdam, the Netherlands

**Keywords:** tuberculosis, mouse, pharmacokinetics, pharmacodynamics, treatment duration

## Abstract

**Background:**

Given the persistently high global burden of tuberculosis, effective and shorter treatment options are needed. We explored the relationship between relapse and treatment length as well as interregimen differences for 2 novel antituberculosis drug regimens using a mouse model of tuberculosis infection and mathematical modeling.

**Methods:**

*Mycobacterium tuberculosis–*infected mice were treated for up to 13 weeks with bedaquiline and pretomanid combined with moxifloxacin and pyrazinamide (BPaMZ) or linezolid (BPaL). Cure rates were evaluated 12 weeks after treatment completion. The standard regimen of isoniazid, rifampicin, pyrazinamide, and ethambutol (HRZE) was evaluated as a comparator.

**Results:**

Six weeks of BPaMZ was sufficient to achieve cure in all mice. In contrast, 13 weeks of BPaL and 24 weeks of HRZE did not achieve 100% cure rates. Based on mathematical model predictions, 95% probability of cure was predicted to occur at 1.6, 4.3, and 7.9 months for BPaMZ, BPaL, and HRZE, respectively.

**Conclusion:**

This study provides additional evidence for the treatment-shortening capacity of BPaMZ over BPaL and HRZE. To optimally use preclinical data for predicting clinical outcomes, and to overcome the limitations that hamper such extrapolation, we advocate bundling of available published preclinical data into mathematical models.

## Teaser

By combining the evaluation of treatment efficacy of anti-tuberculosis drug regimens in a mouse tuberculosis infection model with mathematical modeling, it was found that BPaMZ had a higher treatment-shortening potential than BPaL, compared to the standard HRZE regimen.

With 10 million new cases and 1.5 million deaths in 2018, tuberculosis remains a major global problem [1]. The rise of antimicrobial resistance threatens attempts to reduce the burden of tuberculosis. Multidrug-resistant (MDR) tuberculosis requires a burdensome treatment regimen of ≥9–12 months, compromising treatment adherence [2]. Furthermore, reported treatment success rates are as low as 54% [1]. Until recently, treatment options were even more limited for extensively drug-resistant (XDR) tuberculosis [1]. Increasing attention toward the development of new therapeutic options resulted in the approval of 3 new tuberculosis compounds: bedaquiline, delamanid, and pretomanid. These compounds are being tested in combination with other new, repurposed, or established drugs to accelerate clinical implementation. Two such examples of new, all-oral regimens are bedaquiline and pretomanid combined with either moxifloxacin and pyrazinamide (BPaMZ) or linezolid (BPaL). 

The superior bactericidal and sterilizing capacity of both BPaMZ and BPaL, compared with the first-line regimen, consisting of isoniazid, rifampicin, pyrazinamide, and ethambutol (HRZE), has been demonstrated in preclinical studies [3–5]. These studies indicate that BPaMZ cures *Mycobacterium tuberculosis* infection in mice in a shorter treatment duration than BPaL, which, in turn, achieves cure more quickly than HRZE. In an 8-week phase IIb clinical study, BPaMZ was shown to be safe and effective in patients with rifampicin-resistant tuberculosis [6]. The efficacy of BPaMZ in patients with drug-susceptible tuberculosis or drug-resistant tuberculosis is currently being studied in the SimpliciTB trial (ClinicalTrials registration NCT03338621). BPaL, however, was recently approved by the Food and Drug Administration and the European Medicines Agency as a 6-month regimen for XDR tuberculosis and treatment-intolerant MDR tuberculosis. BPaL was shown to improve treatment options and outcomes for this patient population, with a 90% cure rate observed in the Nix-TB trial [7]. However, exact treatment durations for BPaMZ and BPaL required to achieve desirable cure rates remain to be established through further (pre)clinical studies.

A better understanding of the relationship between treatment duration and treatment outcome is essential to guide recommendations on tuberculosis treatment duration. Because this is costly and time-consuming to assess in clinical studies, preclinical animal studies are usually conducted, and taken together with early-stage clinical trials, the results can guide the design of late-stage clinical trials [8]. However, the translational value of animal studies was critically evaluated when phase III studies investigating the integration of moxifloxacin into first-line regimens failed to reproduce encouraging preclinical results [8–13]. 

Mathematical modeling is one way to extract more information from animal studies, while considering the “3R” principles of replacement, reduction, and refinement [14]. Furthermore, pharmacometric modeling can significantly improve the translation of preclinical data to clinical settings [15]. We recently improved our in vivo experimental design, such that the data are better suited for mathematical modeling [16]. Additional treatment durations were implemented, testing fewer mice per time point. Subsequent in silico simulations enabled continuous linkage of treatment duration and probability of cure. This is an advantage over studies that investigate treatment outcome only at specific time points. 

In the current work, we apply this mathematical model-based approach to evaluate treatment outcomes of BPaMZ, BPaL, and HRZE. We can thereby verify whether the strategy can be applied to other drug regimens too. Moreover, it provides insight into the relationship between treatment duration and cure rates for BPaMZ, BPaL, and HRZE, and allows for efficient comparison between the regimens. As such, results of the present study might pave the way toward bundling of available preclinical data, thereby creating even more robust models that can guide recommendations on optimal clinical tuberculosis treatment duration.

## METHODS

### Animals, Mycobacterial Strain, and Infection

Specified pathogen-free female BALB/c mice, aged 12–13 weeks, were obtained from Charles River. A total of 80 mice per treatment group (29 for pharmacokinetic analysis and 51 for treatment efficacy) were infected with *M. tuberculosis* Beijing VN 2002-1585, as described elsewhere [17]. Briefly, under general anesthesia, animals were infected by intratracheal instillation of 0.96 × 10^5^ (range, 0.88–1.10 × 10^5^) colony-forming units (CFUs), followed by inhalation to ensure formation of bilateral infection. Mice were checked daily and were euthanized when humane end points were reached. The minimal inhibitory concentrations of the compounds for this *M. tuberculosis* strain were determined according to Clinical and Laboratory Standards Institutes guidelines [18]. For bedaquiline, the minimal inhibitory concentration was 0.125 mg/L, for pretomanid, 0.06 mg/L, for linezolid, 0.25 mg/L, and for moxifloxacin, 0.125 mg/L, which were considered susceptible [19]. The strain was susceptible to pyrazinamide, as tested by the BACTEC MGIT-960 system (Becton Dickinson).

### Ethical Approval

Experimental protocols adhered to the rules specified in the Dutch Animal Experimentation Act and were in concordance with the European Union animal directive 2010/63/EU (license nos. 117-14-04 and AVD1010020173687).

### Tuberculosis Drugs

Bedaquiline (supplied by TB Alliance) was formulated every 2 weeks in an acidified (pH 2) 20% (wt/vol) hydroxypropyl-β-cyclodextrin (Kleptose, Roquette) solution. Pretomanid (supplied by TB Alliance) was suspended in a cyclodextrin micelle formulation containing 5% (wt/vol) hydroxypropyl-β-cyclodextrin and 10% (wt/vol) lecithin (ICN Pharmaceuticals). A 100-mg/mL suspension was prepared monthly. Dilutions in distilled water were prepared weekly to achieve desired concentrations. Supplementary File 1 provides additional information on the preparation of pretomanid. Moxifloxacin (BOC Sciences) and pyrazinamide (Sigma-Aldrich) were dissolved together in distilled water by heating to 55ºC. Linezolid (Ambinter) was suspended in a 0.5% (wt/vol) methylcellulose (Sigma-Aldrich) solution in distilled water. All formulations were stored at 4ºC. Drugs were administered together in either the BPaMZ or BPaL combination in a volume of 0.2 mL, once daily by oral gavage, 5 days per week. Drug doses were as follows: bedaquiline, 25 mg/kg; pretomanid, linezolid, and moxifloxacin, 100 mg/kg each; and pyrazinamide, 150 mg/kg.

### Pharmacokinetic Analyses

Drug concentrations, including the *N*-desmethyl bedaquiline metabolite (M2), in mouse serum were quantified after 4 weeks of BPaMZ or BPaL treatment (steady-state drug concentrations). At 0.25, 0.5, 0.75, 1, 1.5, 2, 3, 4, 6, 8, 12, and 24 hours after drug administration 2 mice were sacrificed and blood samples were collected in 2-mL microcentrifuge tubes (Sarstedt ) by orbital sinus bleeding. Samples were allowed to clot for 30 minutes at 4°C, and serum was separated by centrifugation (10 000*g* for 5 minutes). Serum was decontaminated with acetonitrile (Biosolve) at a ratio of 1:3 respectively. After vortexing and centrifugation (10 000*g* for 5 minutes), clear supernatant was transferred into cryotubes. Serum samples were stored at −80°C.

Serum analyte concentrations were assessed using liquid chromatography-tandem mass spectrometry (LC-MS/MS), as described in Supplementary File 2. Briefly, standard, quality control, control, and matrix blank samples in mouse serum were prepared using 1:3 extraction in acetonitrile. Supernatants of standards, controls, and study samples were mixed with acetonitrile with tolbutamide or without internal standard for matrix blanks, and this extract was prepared and used for subsequent analysis. LC-MS/MS analysis of bedaquiline, M2, pretomanid, moxifloxacin, and linezolid was performed on a Shimadzu Nexera X2 liquid chromatograph with a Thermo BetaBasic-4 column (2.1 × 50 mm; 5 µm), coupled to an AB Sciex Triple Quad 5500 mass spectrometer. For pyrazinamide analysis, a Thermo Aquasil C18 column (2.1 × 50 mm; 5 µm) and AB Sciex Triple Quad 6500 mass spectrometer were used. The lower limit of quantification for all compounds was 5 ng/mL. Sample analysis was accepted if quality control sample concentrations were within 20% of the nominal concentration. Data were processed using Analyst 1.6.3 software (Sciex). The maximum drug concentrations and area under the concentration-time curve over 24 hours were determined using noncompartmental analysis in GraphPad Prism 8 software (GraphPad Software).

### Treatment Outcome Assessment

Two weeks after infection, just before the start of treatment, 3 mice per treatment group were euthanized to determine mycobacterial load in lungs and spleen at baseline. The treatment duration ranged from 4 to 11 weeks for BPaMZ, and from 6 to 13 weeks for BPaL, based on results from other preclinical studies [3–5]. Twice weekly, treatment was stopped for 3 mice per group. To assess whether the elapsed treatment duration led to cure, mice were euthanized 12 weeks after treatment completion. as described elsewhere [17]. Lungs and spleen were removed aseptically, homogenized, and serially diluted. Dilutions were cultured on 7H10 Middlebrook agar plates (BD) with activated charcoal to prevent drug carryover, and on plates without charcoal. Because *M. tuberculosis* grew better on charcoal-lacking plates, the mycobacterial load was first assessed by CFU counting on these plates. When no CFUs were detected, charcoal-containing plates were checked to determine whether this was an effect of drug carryover. The lower limit of detection is 11.5 CFUs per lung, calculated from a single colony detected in 200 μL plated from 2.3 mL of lung homogenate per mouse, and 10.5 CFUs per spleen, based on 200 μL from 2.1 mL of spleen homogenate.

### Statistical Analysis

CFU counts were log10-transformed before analysis. An unpaired 2-tailed *t* test was used to compare exposure to bedaquiline, M2, and pretomanid between BPaMZ and BPaL, and mean CFU counts between the treatment groups at the start of treatment. The level of statistical significance was set at α = .05. Statistical analysis was performed using GraphPad Prism software, version 8 (GraphPad Software).

The experimental BPaMZ and BPaL data, together with HRZE data from a previous study with the same experimental protocol [20], were used to build the mathematical model. All data were analyzed simultaneously to allow for evaluation of potential differences between regimens. The model building strategy was described elsewhere [16]. Observed CFU counts in the lungs at 12 weeks after treatment completion were converted to binary outcome values of cure (no CFUs detected) or failure (CFUs detected). Evaluation of different models was based on their objective function value, indicating the likelihood of a model to fit the data, scientific plausibility, parameter uncertainty, and visual predictive checks. 

Model development was performed in 2 steps. First, the relation between probability of cure and treatment duration, regardless of the drug regimen, was described. The starting point was a base model which assumed there is no relation between cure rates and treatment duration. Next, different relations with respect to treatment duration were evaluated, including a linear model, an E_max_ model, and a sigmoidal E_max_ model. Among these, the model that best fitted the experimental data was taken to the second step, which evaluated whether the relation was significantly different between the 3 regimens. In a stepwise approach, various models were fitted to determine whether a given model parameter differed significantly between 1 regimen and the other 2. Only models that significantly lowered the objective function value by >3.84 points (*P* < .05) were further evaluated in combinations.

In the experimental setup, 3 mice were tested by treatment duration in each regimen, limiting cure rates to 0%, 33%, 67%, or 100%. To predict cure rates in the entire range between 0% and 100%, the data set was bootstrapped. Model parameters were then reestimated using 1000 resampled data sets from the observed data with replacement. From the resulting distribution of 1000 parameter estimates, 95% probability of cure and 90% confidence intervals were predicted. The data were analyzed using NONMEM 7.4.3 (ICON) [21]. Visual predictive checks, generated using 1000 simulations, were produced using Xpose [22] and Perl-speaks-NONMEM (PsN) 4.10.0 software [22]. Data management and graphic analysis were performed using R 3.6.3 software [23].

## RESULTS

### Pharmacokinetics in BALB/c Mice

**Figure 1.**
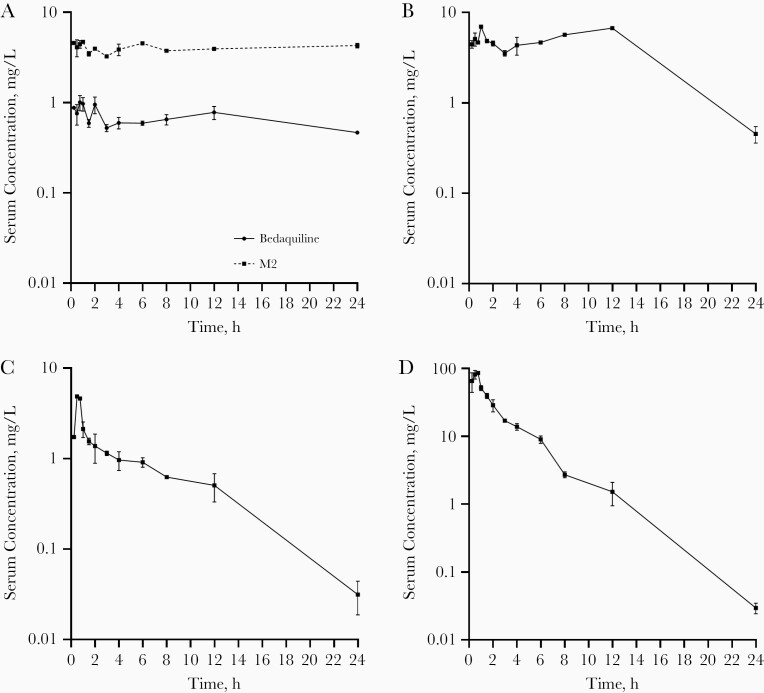
*Mycobacterium tuberculosis*–infected BALB/c mice (n = 2 per time point) were treated 5 times per week for 4 weeks with bedaquiline (25 mg/kg), pretomanid (100 mg/kg), moxifloxacin (100 mg/kg), and pyrazinamide (150 mg/kg). Bedaquiline and its *N*-desmethyl bedaquiline metabolite (M2) (*A*), pretomanid (*B*), moxifloxacin (*C*), and pyrazinamide (*D*) serum concentration-time profiles are plotted as means with ranges (error bars) at various time points after the last drug administration.

**Figure 2.**
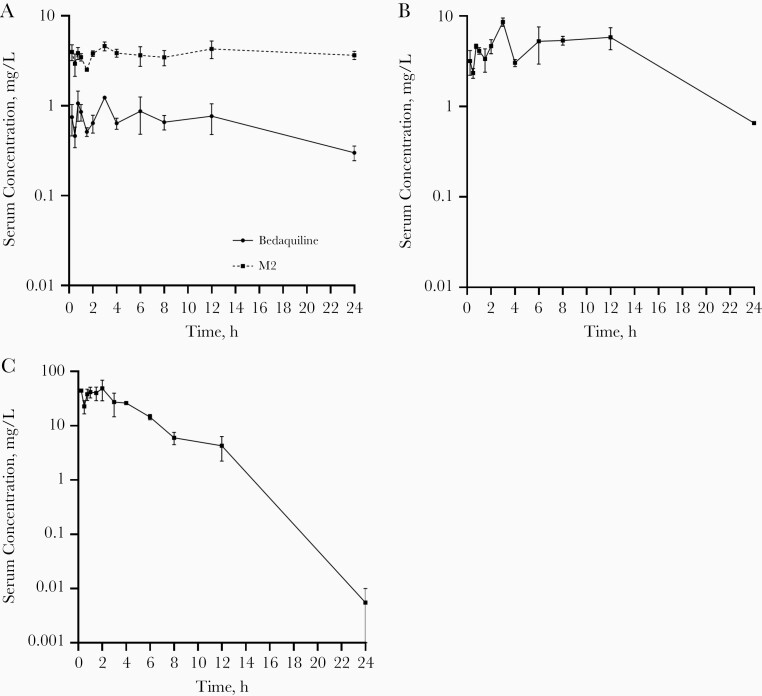
*Mycobacterium tuberculosis*–infected BALB/c mice (n = 2 per time point) were treated 5 times per week for 4 weeks with bedaquiline (25 mg/kg), pretomanid (100 mg/kg), and linezolid (100 mg/kg). Bedaquiline and its *N*-desmethyl bedaquiline metabolite (M2) (*A*), pretomanid (*B*), and linezolid (*C*) serum concentration-time profiles are plotted as means with ranges (error bars) at various time points after the last drug administration.

Serum concentration-time profiles and pharmacokinetic parameters of each drug in BPaMZ and BPaL are shown in Figures 1 and 2, and Table 1. There was no significant difference in exposure to bedaquiline (*P* = .95), M2 (*P* = .67), and pretomanid (*P* = .74), expressed as the area under the concentration-time curve over 24 hours, between BPaMZ and BPaL. Pharmacokinetic parameters that drive treatment efficacy of the tested compounds are in line with findings of previous preclinical studies [4, 24–30].

**Table 1.** Pharmacokinetic Analysis Serum Values by Treatment Regimen (n = 2 per Time Point)

Regimen	Compound (mg/kg)	C_max_, Range, mg/L	AUC_0–24h_, Mean (SEM), mg ⋅ h/L
BPaMZ	Bedaquiline (25)	0.81–1.20	15.52 (1.22)
	*N*-desmethyl bedaquiline	4.55–4.87	95.78 (2.34)
	Pretomanid (100)	6.89–7.03	104.20 (2.44)
	Moxifloxacin (100)	4.78–4.96	15.70 (1.67)
	Pyrazinamide (150)	89.90-81.60	186.80 (9.41)
BPaL	Bedaquiline (25)	1.20–1.26	15.32 (2.75)
	*N*-desmethyl bedaquiline	4.07–5.11	91.61 (9.54)
	Pretomanid (100)	7.70–9.50	99.13 (15.14)
	Linezolid (100)	28.70–68.90	240.00 (28.84)

Abbreviations: AUC_0–24h_, area under the concentration-time curve over 24 hours; BPaL, bedaquiline and pretomanid combined with linezolid; BPaMZ, bedaquiline and pretomanid combined with moxifloxacin and pyrazinamide; C_max_, maximum serum concentration; SEM, standard error of the mean.

### Pharmacodynamic Analysis—In Vivo Experiments

**Figure 3.**
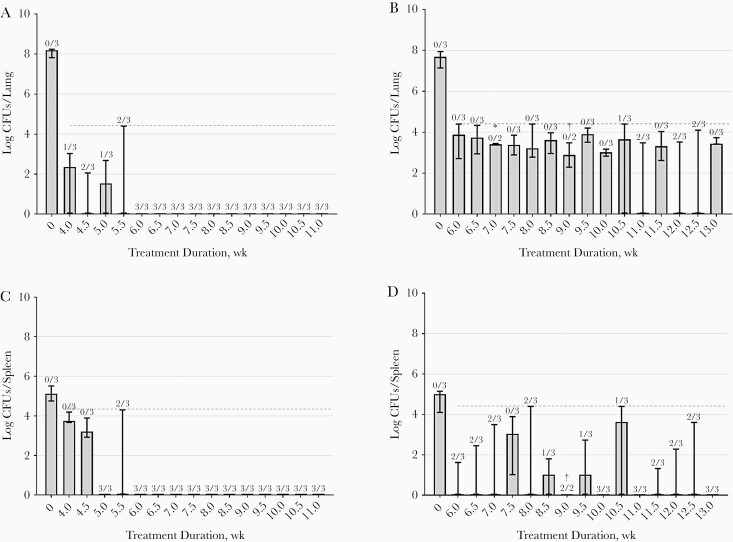
Mycobacterial load in lung (*A, B*) and spleen (*C,* D) expressed as medians with ranges (error bars) of colony-forming units (CFUs) at 12 weeks after different treatment durations. Mice were treated with bedaquiline and pretomanid combined with either moxifloxacin and pyrazinamide (BPaMZ) (*A, C*) or linezolid (BPaL) (*B, D*). Numbers above bars indicate the number of mice with cure relative to the total number examined. Dashed horizontal lines indicate the upper limits of detection (in CFUs). *CFU counting of 1 plate could not be performed owing to contamination. †One mouse reached humane end points and was euthanized before the planned date. Heart and lungs from this mouse were cultured, and no CFUs were recovered on the plates.

Mice tolerated both regimens well, although BPaL-treated mice were considerably more active during the first 3.5 weeks of treatment. One mouse that had received BPaL for 9 weeks required euthanasia on reaching humane end points in the 10th week after treatment completion. The mycobacterial load at 12 weeks after completion of different treatment durations is depicted in Figure 3. Starting inocula were similar in both treatment groups (*P* = .14). Cure was achieved in all mice receiving BPaMZ for ≥6 weeks, with all mice having culture-negative lungs after 12 weeks after treatment completion. In the BPaL group, cure rates exceeding 0% (culture-negative lungs in ≥1 of the 3 mice) were observed after 10.5, 11.0, 12.0, and 12.5 weeks of treatment. Nevertheless, the maximum treatment duration of 13 weeks did not achieve cure in all mice. Mycobacterial loads in the spleen showed similar patterns. HRZE treatment did not reach 100% cure rates. At 12 weeks after the maximum treatment duration of 24 weeks, 1 of the 3 mice had culture-positive lungs [20].

### Pharmacodynamic Analysis—Mathematical Modeling

The final model was a sigmoidal E_max_ relation regarding probability of failure (Pr_failure_) and probability of cure (Pr_cure_) in relation to treatment length and regimen, as follows: 

Pr_failure_ = 1 − Pr_cure_ = Pr_base ⋅_ [1 − [(E_max _⋅ Length^γ^)/T_50_^γ^ + Length^γ^)]],

where E_max_ is the maximum probability of cure fixed to 1, T_50_ is the regimen-specific treatment duration at which 50% of E_max_ is achieved, Pr_base_ is the probability of failure with no treatment, and γ is the Hill factor parameter that controls the shape and steepness of the E_max_ curve.

**Figure 4.**
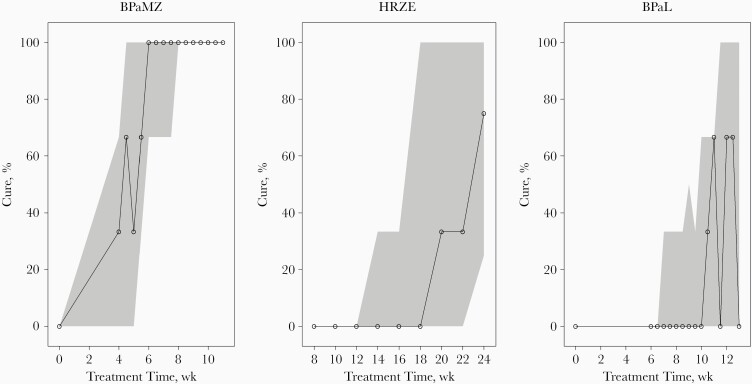
Visual predictive checks of the final model for each regimen, using 1000 simulations. Solid lines with open circles represent observed probabilities of cure; the shaded areas, 95% confidence intervals of the predicted cure rates. Abbreviations: BPaL, bedaquiline and pretomanid combined with linezolid; BPaMZ, bedaquiline and pretomanid combined with moxifloxacin and pyrazinamide; HRZE, isoniazid, rifampicin, pyrazinamide, and ethambutol.

**Figure 5.**
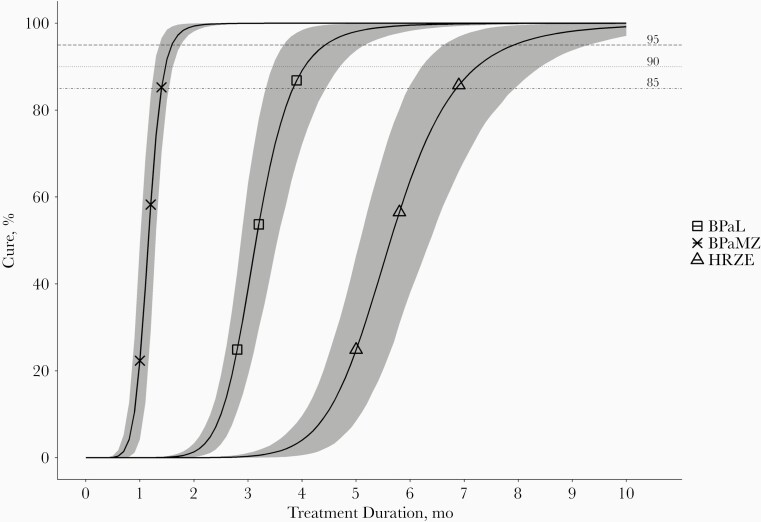
Model-predicted cure rates after different treatment durations for the 3 treatment regimens, using 1000 bootstraps of the original data set and with reestimation using the final model. The lines resemble mean probabilities of cure, and gray-shaded areas, 90% confidence intervals of the predictions from the 1000 distributions of the reestimated model parameters. Abbreviations: BPaL, bedaquiline and pretomanid combined with linezolid; BPaMZ, bedaquiline and pretomanid combined with moxifloxacin and pyrazinamide; HRZE, isoniazid, rifampicin, pyrazinamide, and ethambutol.

In the first step of model building, an E_max_ model was identified to best describe the relation between probability of cure and treatment duration. A sigmoidal E_max_ model provided a significantly lower objective function value but yielded scientifically implausible estimates of E_max_ and γ. Subsequently, the impact of the regimens on the probability of cure was explored in all model parameters. The T_50_ parameter was significantly different for the 3 regimens. When the difference in T_50_ between the regimens was included in the model, a sigmoidal E_max_ relation between treatment duration and probability of cure was reevaluated and determined to best fit the data. Including a regimen-specific γ parameter gave no statistically significant difference for any of the regimens. Therefore, 1 γ parameter was deemed sufficient. Final model parameter estimates are presented in Table 2. Visual predictive checks of the final model are depicted in Figure 4. Predicted treatment durations required to achieve certain probabilities of cure are plotted in Figure 5. The model predicted that 95% probability of cure was reached after 1.6 months for BPaMZ, while this was 4.3 months for BPaL and 7.9 months for HRZE.

**Table 2.** Parameter Estimates of the Final Mathematical Model

Parameter	Final Estimate	RSE, %^a^	Mean Estimate (90% CI)^b^
Pr_base_	Fixed to 1	–	Fixed to 1
E_max_	Fixed to 1	–	Fixed to 1
T_50_, mo			
BPaMZ	1.15	7.58	1.15 (1.01–1.30)
HRZE	5.65	6.51	5.67 (4.98–6.36)
BPaL	3.16	6.14	3.17 (2.84–3.51)
γ	9.15	18.66	10.1 (6.41–13.84)

Abbreviations: γ, Hill factor parameter; BPaL, bedaquiline and pretomanid combined with linezolid; BPaMZ, bedaquiline and pretomanid combined with moxifloxacin and pyrazinamide; CI, confidence interval; E_max_, maximum probability of cure fixed to 1; HRZE, isoniazid, rifampicin, pyrazinamide, and ethambutol; Pr_base_, probability of failure with no treatment; RSE, relative standard error; T_50_, treatment duration at which 50% of E_max_ is achieved.

^a^Relative standard error on the approximate standard deviation scale as obtained from the covariance step in NONMEM.

^b^Mean estimate and 90% CIs were obtained by bootstrapping the data set, followed by reestimation with the final model (n = 1000).

^c^T_50_ differed significantly between the regimens.

## DISCUSSION

In the current study, treatment with BPaMZ led to a rapid decline in mycobacterial load, and achieved cure in all mice after 6 weeks of treatment. Its treatment-shortening capacity is superior to that of BPaL and HRZE, as the mathematical model predicts that 95% probability of cure is reached after 1.6 months for BPaMZ, 4.3 months for BPaL, and 7.9 months for HRZE. The order of efficacy is consistent with other mouse tuberculosis studies, in which 1.5–2 months of BPaMZ-treatment was sufficient for cure [3, 5], while 3 months were needed for BPaL [4, 5], and ≥6 months for HRZE [17].

Compared with other mouse tuberculosis models, BPaL performance was unexpectedly low. It could be speculated that the discrepancy in BPaL efficacy is a consequence of the different *M. tuberculosis* strains used, as was previously shown for HRZE [17, 31]. We used a Beijing strain, known for its virulence and clinical relevance [32–35], while other studies used the H37Rv strain [4, 5]. The different treatment outcomes highlight the importance of using various *M. tuberculosis* strains to assess treatment efficacy. It is noteworthy that in the context of containing regimens containing bedaquiline and pretomanid, linezolid shows differential efficacy against H37Rv versus Beijing strains, whereas moxifloxacin- and pyrazinamide-containing regimens achieve similar efficacy against both strains [3, 5]. It could be that drug interactions within BPaL are more favorable in treating H37Rv than in treating Beijing strains. 

This interpretation is strengthened in a finding by Bigelow et al [36] that drug interactions within BPaL varied according to *M. tuberculosis* strain. In mice infected with HN878 (belonging to the W-Beijing family), both bedaquiline-pretomanid and bedaquiline-linezolid performed better than BPaL, while against H37Rv, BPaL was the best-performing drug combination in that study. What mechanisms underlie such strain-dependent drug interactions is unclear. The reasons for the apparent superiority of BPaMZ over BPaL observed in this and other preclinical studies are not yet elucidated. In vitro interaction between bedaquiline and pyrazinamide is known to be synergetic [37, 38], whereas interaction between bedaquiline and linezolid was shown to be additive [39], indifferent [36], or even antagonistic [40]. However, we find that exposure to bedaquiline, its M2 metabolite, and pretomanid are similar for BPaMZ and BPaL, which argues against drug-drug interactions as drivers of the rank order in treatment efficacy.

The present study confirms that the improved experimental setup and mathematical modeling, as we introduced elsewhere [16], can shed light on the relationship between treatment duration and treatment outcome, and facilitates efficient comparison between regimens. This setup differs from conventional mouse tuberculosis studies, because treatment efficacy is assessed 12 weeks after treatment completion (sterilizing activity) but not directly after treatment completion (bactericidal activity). Moreover, sample sizes are smaller, with only 3 mice euthanized per treatment duration [3–5, 16]. Yet this design with additional treatment durations and small sample sizes still had power to evaluate treatment differences using mathematical modeling, as it was found to be sufficient to detect a 50% difference in potency between regimens and reached high precision in model parameters [16].

In our modeling approach, we assumed that all regimens can eventually result in 100% cure (E_max_ fixed at 100%). As such, not all regimens in the mouse experiments need to reach 100% cure, as was the case for HRZE and BPaL. Especially for new regimens it can be difficult to select optimum treatment durations in the experimental design. Mathematical modeling adds value, as the probability of cure can be predicted for all regimens as long as one regimen provides information about the relationship between treatment length and almost-complete cure, together with the assumption that only T_50_ differs between the regimens. The modeling approach could also estimate E_max_ for regimens that never reach 100% cure. However, in this case, the experimental design would need to include data on maximal cure.

Our combined experimental-mathematical model approach provides guidance on treatment durations needed to reach certain cure rates. The Nix-TB trial, a phase III study that investigated the efficacy of BPaL in patients with MDR or XDR tuberculosis, demonstrated a 90% treatment success rate after 6 months of treatment [7]. It is tempting to speculate that shorter treatment durations might be sufficient, considering the predicted 95% probability of cure in mice at 4.3 months. However, several limitations in our model should be considered when extrapolating results to the clinical situation.

First, BALB/c mice develop cellular granulomas with minimal necrosis on tuberculosis infection [41], whereas necrotizing, caseous lesions are a hallmark of human tuberculosis. The distinct environmental conditions in caseous lesions (eg, hypoxia, more neutral pH) influence local drug effects [42]. Bedaquiline, pretomanid, and moxifloxacin are reported to accumulate in cellular regions rather than in necrotic areas of granulomas [25, 42, 43], while pyrazinamide and linezolid seem to diffuse equally well through these compartments [42, 44]. 

This characteristic of pyrazinamide and linezolid is perhaps less clearly expressed in our mouse model, since the granulomas are mostly cellular instead of necrotizing. Hence, these diffusion patterns might imply that BPaMZ could generate more favorable results than BPaL in our model versus models with necrotic granulomas. It should be noted that although the aforementioned studies use elegant methods to approximate drug concentrations at the infection site, exposure-response relationships based on such data should be interpreted with caution [45]. This limitation is (partly) addressed by using C3HeB/FeJ mice, in which lung disease on tuberculosis infection more closely resembles human tuberculosis [25]. As BPaMZ also seems to outperform BPaL and HRZE in C3HeB/FeJ mouse tuberculosis models [5, 36, 46], the impact of different lung pathology on the translational value of our results is probably modest.

Second, whether a mouse tuberculosis model represents acute, subacute, or chronic infection depends on the activity of the adaptive immune system. This depends on the time window between infection and treatment initiation [8]. In acute infection models, bacilli replicate logarithmically, which is suitable for assessing a regimen’s bactericidal capacity. Slowly or nonreplicating bacilli are present in chronic infection models, which are effective for determining sterilizing activity. The present model resembles subacute infection [17], characterized by a more heterogeneous mycobacterial population [8]. Since a complex spectrum of lung lesions is present in patients with tuberculosis [47], including data from both acute and chronic preclinical infection models could enrich the input of the mathematical model.

In conclusion, with the current study we provide additional evidence in favor of the treatment-shortening capacity of BPaMZ over BPaL, and BPaL over HRZE. To enable the optimal use of preclinical data and to overcome the limitations that hamper extrapolation of animal data to humans, we advocate bundling of available preclinical data into mathematical models. As such, the predictive value of mathematical models could be enhanced in their ability to guide decision making on treatment durations, which is needed to achieve desirable cure rates in patients with tuberculosis.

## Supplementary Material

jiab101_suppl_Supplementary_Data

## Notes

***Acknowledgments.*** The authors thank Sanne van den Berg, Marian ten Kate, and Heleen van der Spek for their technical assistance.

***Financial support.*** This work was supported by the TB Alliance with funding from Australia’s Department of Foreign Affairs and Trade, the Bill & Melinda Gates Foundation (grant OPP1129600), Germany’s Federal Ministry of Education and Research through the KfW, Irish Aid, the National Institute of Allergy and Infectious Disease, the Netherlands Ministry of Foreign Affairs, the United Kingdom Department for International Development, and the US Agency for International Development.

***Potential conflict of interest.*** All authors: No reported conflicts. All authors have submitted the ICMJE Form for Disclosure of Potential Conflicts of Interest. Conflicts that the editors consider relevant to the content of the manuscript have been disclosed.
