# Supplementary material for: Predictive Modeling to Study the Treatment-Shortening Potential of Novel Tuberculosis Drug Regimens, Toward Bundling of Preclinical Data
Source: J Infect Dis. 2021 Feb 19;225(11):1876–85. doi: 10.1093/infdis/jiab101 (PMC9159334; doi:10.1093/infdis/jiab101)
Supplement: jiab101_suppl_Supplementary_Data [file jiab101_suppl_supplementary_data.docx]

**Predictive modeling to study the treatment-shortening potential of novel tuberculosis drug regimens, towards bundling of preclinical data**

Saskia E. Mudde, Rami Ayoun Alsoud, Aart van der Meijden, Anna M. Upton, Manisha U. Lotlikar, Ulrika S.H. Simonsson, Hannelore I. Bax, Jurriaan E.M. de Steenwinkel

**Online supplement**

**Supplementary file 1.**

Pretomanid was first suspended in 10% (w/v) hydroxypropyl-beta-cyclodextrin (HPbCD) (Kleptose, Roquette BV, Hoofddorp, The Netherlands) and stirred for 24 hours on a magnetic stirrer to form a pretomanid-HPbCD complex. After stirring, the suspension was sonicated with a thick probe at ± 8% amplitude for 10 minutes. Next, an equal volume 20% (w/v) lecithin solution was added to reach a final concentration of 50 mg/ml. The suspension was stirred for 10 minutes on a magnetic stirrer to form a lipid bilayer around the complex. Subsequently, the suspension was sonicated at 10% amplitude for 30 minutes and was diluted in water to the desired end concentration. The suspension was stirred daily for 15 minutes before addition to the BPaL cocktail.

**Supplementary file 2.**

*Standard curves and quality controls*: Neat standard stock solutions for all compounds were prepared at 1.0 mg/mL in dimethylsulfoxide (DMSO)/methanol (MeOH) (1:1). To set up calibration standards and quality controls (QC), stock solutions were serially diluted in mouse serum K2 EDTA for bedaquiline, *N*-monodesmethyl bedaquiline, pretomanid, moxifloxacin, and linezolid, and in blank mouse serum containing 10% formic acid in the case of pyrazinamide. The standard curve ranged from 5 to 5,000 ng/mL. Four concentrations were used for QC, with 15 ng/mL for the low QC, 250 ng/mL for middle QC, 4,000 ng/mL for high QC, and 25,000 ng/mL for dilution QC.

*Sample Extraction Procedures*: Briefly, 50 μl of blank matrices, control samples, standards, and QC samples were combined with 150 μl acetonitrile (ACN), paralleling the decontamination of the study samples. For all analytes except pyrazinamide, after vortexing and centrifugation, 25 μl of supernatant from the previous step or from decontaminated study samples was combined with 200 μl ACN containing internal standard (100 ng/mL tolbutamide), except for matrix blanks where ACN without internal standard was used. The mixtures were again vortexed and centrifuged. To 25 μl of the extracted supernatant, 250 μl ACN:water 30:70 was added for analysis of bedaquiline, *N*-desmethyl bedaquiline, and pretomanid. In the case of moxifloxacin and linezolid analysis, 50 μl of the extracted supernatant was combined with 200 μl of deionized water. For the extraction procedure of pyrazinamide, after the first extraction step, 50 μl of supernatant was mixed with 50 μl ACN containing 5 ng/mL tolbutamide, or ACN without internal standard for matrix blanks. Following vortexing and centrifugation, the resulting solution was evaporated to dryness under a steady stream of nitrogen at 50°C, after which 150 μl MeOH/water (20:80) was added. All mixtures were then vortexed and centrifuged before storage at 2-8°C or further analysis by HPLC set to 4°C.

*LC-MS/MS analysis*: For the mobile phase A 0.1% formic acid in water was used (with 10 mM ammonium formate in the case of moxifloxacin and linezolid) and 0.1% formic acid in ACN for the mobile phase B. Injection volumes were either 10 μl (bedaquiline, *N*-desmethyl bedaquiline, pretomanid) or 5 μl (moxifloxacin, linezolid, pyrazinamide). Quantification of the analytes was performed using multiple-reaction monitoring (MRM) of parent/product transitions in electrospray positive-ionization mode. The MRM transitions used were as follows: bedaquiline (555.2/58.1), *N*-monodesmethyl bedaquiline (541.2/480.2), pretomanid (360.2/175.1), moxifloxacin (402.3/364.2), pyrazinamide (124.1/81.1), linezolid (338.2/235.0), and tolbutamide (271/155.1).
